# Exploring the Perspectives of Patients Living With Lupus: Retrospective Social Listening Study

**DOI:** 10.2196/52768

**Published:** 2024-02-02

**Authors:** Erica Spies, Thomas Andreu, Matthias Hartung, Josephine Park, Paul Kamudoni

**Affiliations:** 1 EMD Serono Billerica, MA United States; 2 Semalytix GmbH Bielefeld Germany; 3 The Healthcare Business of Merck KGaA Darmstadt Germany

**Keywords:** systemic lupus erythematosus, SLE, cutaneous lupus erythematosus, CLE, quality of life, health-related quality of life, HRQoL, social media listening, lupus, rare, cutaneous, social media, infodemiology, infoveillance, social listening, natural language processing, machine learning, experience, experiences, tagged, tagging, visualization, visualizations, knowledge graph, chronic, autoimmune, inflammation, inflammatory, skin, dermatology, dermatological, forum, forums, blog, blogs

## Abstract

**Background:**

Systemic lupus erythematosus (SLE) is a chronic autoimmune inflammatory disease affecting various organs with a wide range of clinical manifestations. Cutaneous lupus erythematosus (CLE) can manifest as a feature of SLE or an independent skin ailment. Health-related quality of life (HRQoL) is frequently compromised in individuals living with lupus. Understanding patients’ perspectives when living with a disease is crucial for effectively meeting their unmet needs. Social listening is a promising new method that can provide insights into the experiences of patients living with their disease (lupus) and leverage these insights to inform drug development strategies for addressing their unmet needs.

**Objective:**

The objective of this study is to explore the experience of patients living with SLE and CLE, including their disease and treatment experiences, HRQoL, and unmet needs, as discussed in web-based social media platforms such as blogs and forums.

**Methods:**

A retrospective exploratory social listening study was conducted across 13 publicly available English-language social media platforms from October 2019 to January 2022. Data were processed using natural language processing and knowledge graph tagging technology to clean, format, anonymize, and annotate them algorithmically before feeding them to Pharos, a Semalytix proprietary data visualization and analysis platform, for further analysis. Pharos was used to generate descriptive data statistics, providing insights into the magnitude of individual patient experience variables, their differences in the magnitude of variables, and the associations between algorithmically tagged variables.

**Results:**

A total of 45,554 posts from 3834 individuals who were algorithmically identified as patients with lupus were included in this study. Among them, 1925 (authoring 5636 posts) and 106 (authoring 243 posts) patients were identified as having SLE and CLE, respectively. Patients frequently mentioned various symptoms in relation to SLE and CLE including pain, fatigue, and rashes; pain and fatigue were identified as the main drivers of HRQoL impairment. The most affected aspects of HRQoL included “mobility,” “cognitive capabilities,” “recreation and leisure,” and “sleep and rest.” Existing pharmacological interventions poorly managed the most burdensome symptoms of lupus. Conversely, nonpharmacological treatments, such as exercise and meditation, were frequently associated with HRQoL improvement.

**Conclusions:**

Patients with lupus reported a complex interplay of symptoms and HRQoL aspects that negatively influenced one another. This study demonstrates that social listening is an effective method to gather insights into patients’ experiences, preferences, and unmet needs, which can be considered during the drug development process to develop effective therapies and improve disease management.

## Introduction

Systemic lupus erythematosus (SLE) is an autoimmune inflammatory disease affecting multiple systems in the body; it is characterized by fluctuating symptoms and periods of exacerbation and remission [1-3]. The most common symptoms of SLE include fatigue, skin rashes, fever, and joint pain or swelling [3]. SLE has several phenotypes and clinical manifestations involving various organs, including the joints, skin, kidneys, and organs of the neurological or hematological systems [1,2]. Cutaneous lupus erythematosus (CLE) can manifest as either a feature of SLE or an independent skin ailment [4,5]. The most prevalent symptoms of CLE include rashes, hair loss, blood vessel inflammation, ulcers, and increased sensitivity to light [6,7]. Living with these symptoms can be physically debilitating for patients and can disrupt their family, social, and professional life, thereby negatively impacting their health-related quality of life (HRQoL) [8,9].

Globally, SLE and CLE are estimated to have incidence rates of 5.1 and 4.3 per 100,000 person-years, respectively [10,11]. These rates vary widely according to demographic factors such as ethnicity, sex, and socioeconomic status, with a higher incidence in women, African Americans and other non-White populations [10,11]. The current treatment options for SLE include immunosuppressive agents, new biological therapies, and combination therapies of biologicals with immunosuppressive and immunomodulating agents [12]. Despite recent advances in therapeutic strategies, most of the current medications available for the treatment of lupus provide only symptomatic relief and are frequently associated with undesirable side effects [13,14].

Considering the high clinical variability, inadequate treatment options, and poor HRQoL of patients, understanding the perspectives and experiences of patients with SLE and CLE may be critical for developing effective therapies and improving disease management [13-15]. In the context of drug development, regulatory bodies and health care decision makers are emphasizing patient-focused drug development, which involves actively seeking and incorporating patients’ perspectives when designing interventions that can meet their needs, improve outcomes, and enhance the overall patient experience [16]. The increased presence and active engagement of patients on digital platforms, specifically social media, can serve as a source for understanding their needs, treatment experiences, and the factors affecting treatment decisions in real-world scenarios [15]. Additionally, data regarding the experiences of patients living with the disease can potentially influence key decisions and activities during drug development [16].

Data regarding the experiences of individuals living with diseases can be obtained through various sources, such as observational studies, interviews, focus groups, and patient advisory boards. Social listening is a promising newer approach that complements traditional methods of data collection by providing additional insights into patients’ perspectives beyond clinical settings [17]. Furthermore, this method captures the voices of vulnerable and difficult-to-reach populations who may otherwise not participate in clinical or epidemiological studies, thereby providing an opportunity for data triangulation [17,18]. In this social listening study, we explored the experience of patients living with lupus (SLE and CLE), as discussed in web-based social media platforms, such as blogs and forums, by searching for posts regarding disease burden, HRQoL impacts, treatment experience, and unmet needs.

## Methods

### Study Design

#### Overview

**Figure 1:**
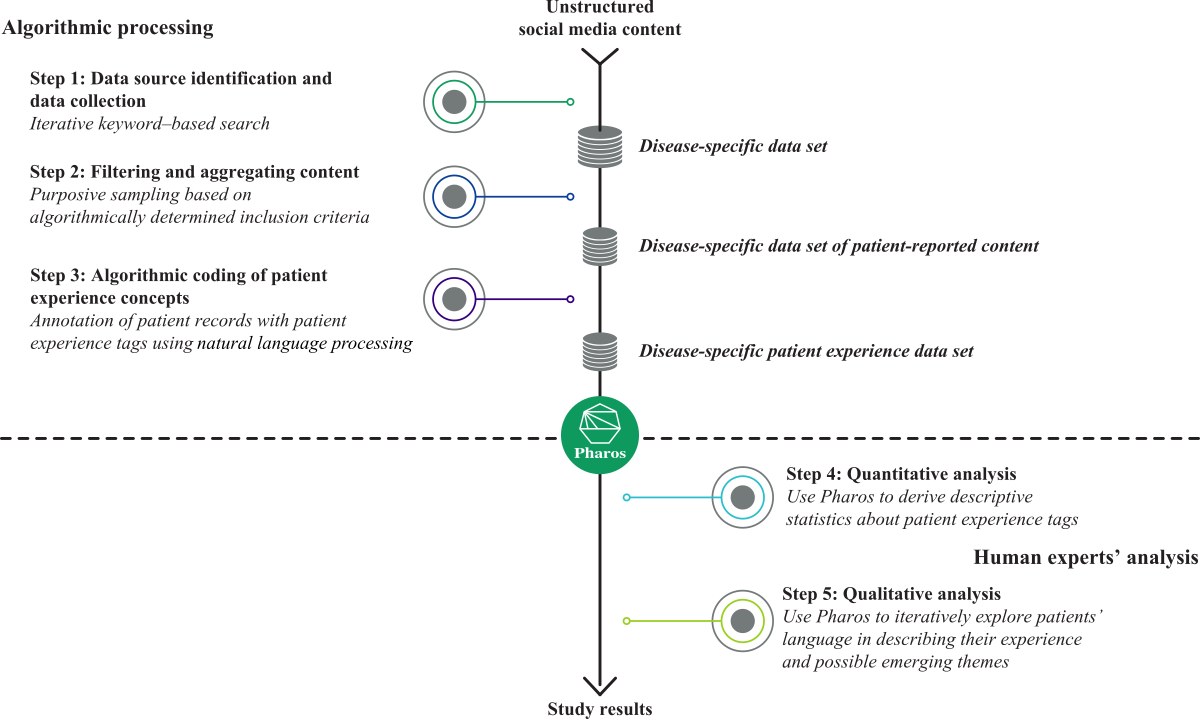
Overview of the study methodology.

This retrospective exploratory social listening study was conducted between October 2019 and January 2022 across 13 publicly available English-language social media platforms (Multimedia Appendix 1). The processes of data identification, collection, and analysis involved have been previously presented [19-22]. The process involved algorithmic processing steps (1-3) and research steps (4 and 5) performed by human analysts (Figure 1). A glossary of terms are provided in Multimedia Appendix 2.

#### Data Source Identification and Data Collection (Step 1)

Identifying data sources was the first step, followed by data collection. Lupus-related keywords, defined and revised by human domain experts, were searched in SocialGist, a third-party search engine providing access to social media sites through an application programming interface search engine, to detect websites hosting relevant lupus-specific content. The following keywords were used for the search: “lupus,” “lupus” AND “systemic” AND “cutaneous” AND “subacute” AND “erythematosus” AND “autoimmune” AND “disease,” “SLE,” and “CLE.” All posts from relevant websites found through the searches were retrieved and collected in Pharos, a proprietary data visualization and analysis platform from Semalytix GmbH.

#### Filtering and Aggregating Content (Step 2)

Filtering and aggregating the content based on algorithmically determined inclusion criteria was the second step. Posts collected in step 1 were aggregated by unique authors, and all posts by the same author were collapsed into 1 author-specific record. Based on algorithmically determined inclusion criteria, only records classified as having been authored by a patient with lupus were retained. Posts authored by nonpatients, such as caregivers and health care providers, and those involving other types of documents, such as journal papers and press releases, were excluded. Throughout this process, human language engineers and data labeling specialists carefully monitored the patient classifications to ensure accuracy and reliability.

#### Algorithmic Coding of Patient Experience Concepts (Step 3)

Patient experience themes were algorithmically coded in the study’s third step. All lupus-specific patient records from step 2 were algorithmically annotated with patient experience tags using natural language processing. Patient experience themes mentioned in texts related to disease burden, HRQoL, and treatment experiences were added to patient records as semantic tags. Individual aspects (facets) of HRQoL were investigated based on the taxonomy provided by the World Health Organization Quality of Life instrument [23], which served as a guide for developing the inventory of patient experience tags used in this study. Human language engineers and data labeling specialists monitored, cross-validated, and adapted the algorithms as needed. A subject matter expert reviewed the study’s overall approach and medical content.

#### Quantitative Analysis (Step 4)

Quantitative data analysis was involved in the fourth step, wherein analysts used the Pharos Patient Experience Platform to generate descriptive statistics regarding patient experience tags. The statistics provided insight into the magnitude of individual patient experience variables, the differences between them, and the associations among the algorithmically tagged variables.

#### Qualitative Analysis (Step 5)

Qualitative data analysis was involved in the fifth step. Human analysts used Pharos to investigate the relationships between tags, such as symptoms and outcomes or symptoms and impacts, as well as the language patients used to describe their experiences on social media to identify any emerging themes. The results of the qualitative analysis were used to identify themes in patient language, connections to tagged variables, potential connections between themes, emerging themes, and reorganized themes.

### Study Population

Authors of posts who self-identified as patients living with lupus or any of its subtypes (SLE and CLE; algorithmically identified) were included in this study. All posts by the same user were aggregated into a unique patient record for further data analysis. A machine learning algorithm classified authors as patients based on the language used in their self-reported posts. The algorithm was specifically designed to differentiate expressions that indicated that a patient had lupus (“I have lupus” or “I was diagnosed with SLE in 2020”) from those that were ambiguous.

### Study Outcomes

Posts from social media platforms were analyzed to investigate symptom burden, impact of lupus on HRQoL, treatment experience, and unmet needs. The related outcomes are described in Table 1. These outcomes were investigated in the SLE as well as CLE-related posts, depending on the robustness of the data.

**Table 1:** Outcomes investigated in the study.

Aspects	Outcomes
Symptom burden	Patient-reported symptoms Most burdensome symptoms^a^ Areas of involvement (body parts) Photosensitivity and body image^a^ Sleep disorders Comorbidities^a^
Overall HRQoL^b^ impact	Psychological well-being (positive feelings and negative feelings) Physical well-being^a^ (mobility^a^, activities of daily living^a^, recreation and leisure, and role participation^a^) Impact and functioning (including parental care, burden to or on others, and economic or working capability impacts)^a^
Treatment experience	Current treatment options (nonpharmacological and pharmacological interventions) Treatment options and HRQoL aspects based on treatment^a^ Level of satisfaction with current treatment options^a^ Diagnostic delay and health care availability Coping mechanisms

^a^Owing to low sample size or insufficient data, these outcomes were not analyzed in the cutaneous lupus erythematosus population.

^b^HRQoL: health-related quality of life.

### Ethical Considerations

Research using data from social media can present ethical challenges owing to the limited guidance on the participants’ consent and anonymity [24]. The data analyzed in this study were obtained from publicly available sources. Identities of the patient posts were appropriately anonymized while ensuring that the data answered specific research questions. To ensure personal data protection, a strict General Data Protection Regulation–compliant process was adopted. Please refer to Multimedia Appendix 3 for further details on the appropriate measures implemented to ensure personal data protection. This study was exempt from ethical review because it examined retrospective publicly available data from a sizeable number of patients.

## Results

### Patients

During the initial data collection (step 1), a total of 76,538 lupus-related posts were collected, of which 45,554 posts from 3834 patients were included in the lupus-specific data set based on the algorithmically determined inclusion criteria. Among these patients, 1925 (with 5636 posts) and 106 (with 243 posts) patients were identified as having SLE and CLE, respectively. In the population with SLE, sex information was available for 583 patients, and the female-to-male ratio was 9:1. Age data were available for 402 patients, with 76.7% (n=308) aged <60 years.

In the following sections, we report the findings regarding the population with SLE followed by the population with CLE. The study results are structured under 3 primary sections: disease burden as reported by patients, overall HRQoL, and treatment experiences reported for both patient populations separately.

### Disease Burden in Patients With SLE

#### Overview

Disease burden was evident among patients with SLE as indicated by a total of 2029 patient mentions across different social media platforms. Disease burden was quantified in terms of the symptoms that were patient-reported, most burdensome, their severity, areas of involvement (body parts), and comorbidities.

#### Patient-Reported Symptoms

The most frequently reported symptoms (n=2029 patients) were pain, fatigue, and rashes. Some patients were more specific regarding the type of pain they experienced, including arthralgia, cephalgia, and arthritis. Other less frequently (<10% [203 patients]) reported symptoms included anxiety, clinical depression, alopecia, and pyrexia (Table 2). In total, 20 patients (in approximately 30 posts) reported experiencing photosensitivity, with excessive sun exposure leading to flare-ups and worsening of symptoms such as joint pain, weakness, and fatigue.

**Table 2:** Symptoms most frequently reported by patients with SLE^a^ (n=2029^b^ patient mentions from 5636 posts) and CLE^c^ (n=118^b^ patient mentions from 244 posts).

Symptoms or signs	Proportion of patients, n (%)	
	SLE	CLE
Pain	610 (30.1)	25 (21.2)
Fatigue	386 (19.2)	19 (16.1)
Rash	239 (11.8)	27 (22.9)
Arthralgia	184 (9.1)	10 (8.5)
Anxiety	150 (7.4)	7 (5.9)
Cephalgia	143 (7.1)	4 (3.4)
Clinical depression	98 (4.8)	—^d^
Arthritis	78 (3.8)	—
Alopecia	71 (3.5)	11 (9.3)
Pyrexia	70 (3.4)	—
Scar	—	7 (5.9)
Photosensitivity	—	4 (3.4)
Hypothyroidism	—	4 (3.4)

^a^SLE: systemic lupus erythematosus.

^b^Indicates that patients were counted more than once if they discussed more than one symptom in a post.

^c^CLE: cutaneous lupus erythematosus.

^d^Not available.

Although sleep disturbances were not among the top 10 patient-reported symptoms, it was found to significantly impact the HRQoL of patients with SLE. Among the 114 patients discussing “sleep and rest” topics, over half of the patients (n=70) reported experiencing recurrent insomnia since many years. Nighttime sleep disturbances and daytime napping were common, with some patients stating that insomnia or a hyperactive mood was an early sign of disease flare-ups. Patients reported that insomnia was also linked to the side effects of certain medications taken for their lupus, such as hydroxychloroquine, methotrexate, and prednisone, whereas medications such as cannabidiol and nonpharmacological interventions including exercise, meditation, and acupuncture had a positive impact on sleep maintenance disorders. Quotations of patients with SLE describing their symptoms are provided in Multimedia Appendix 4.

#### Most Burdensome Symptoms

The most burdensome symptoms were identified by analyzing the degree of severity with which a patient with SLE was affected based on linguistic cues provided by them. The most burdensome symptoms included pain (general pain: 289/870, 33.2%; arthralgia: 59/228, 25.9%; and cephalgia: 65/154, 42.2%), fatigue (192/474, 40.5%), and anxiety (51/193, 26.4%). Other symptoms that were perceived burdensome included clinical depression (30/128, 23.4%), photosensitivity (5/33, 15%), arthritis (12/99, 12.1%), hypothyroidism (2/36, 5.6%), and antiphospholipid syndrome (4/79, 5.1%). Of note, it is possible that the same patient may have reported more than one symptom or sign.

#### Areas of Involvement

The most frequently reported areas of involvement (n=1778 patients) were the skin (n=224, 12.6%), face (n=224, 12.6%), and eyes (n=197, 11.1%; Multimedia Appendix 5).

#### Comorbidities

In addition to the symptoms from the main indication, patients with SLE reported experiencing multiple comorbidities (n=488 patients). The most frequently (>5% [24 patients]) reported comorbidities were major depressive disorder (n=98, 20.1%), fibromyalgia (n=66, 13.5%), infection (n=62, 12.7%), dry eye syndrome (n=50, 10.2%), rheumatoid arthritis (n=40, 8.2%), and hypothyroidism (n=34, 7%).

### Overall HRQoL in Patients With SLE

#### Overview

To investigate the HRQoL of patients, the frequency of mentions and the perceived importance of issues related to HRQoL facets were analyzed. Social networking communities discussed several aspects of HRQoL, as evidenced by 2199 patient mentions and 2138 posts accounting to 37.9% (2138/5636) of all posts analyzed in this study. These posts on HRQoL by patients with SLE contained descriptions of how they perceived the most burdensome symptoms and the impact on their daily lives. The most commonly discussed HRQoL aspects based on the total number of patients mentioning any HRQoL facet (n=2199 patient mentions) were “negative feelings” (n=521, 23.7%) and “recreation and leisure” (n=314, 14.3%). Other commonly discussed HRQoL aspects included “health care availability,” “positive feelings,” “mobility,” “energy and motivation,” “cognitive capabilities,” “sleep and rest,” “work capacity,” and others (Table 3).

**Table 3:** Distribution of HRQoL^a^ aspects among patients with SLE^b^ (n=2199^c^ patient mentions from 5636 posts) and CLE^d^ (n=78^c^ posts from n=47 patients).

HRQoL aspects	Proportion of patients, n (%)	
	SLE	CLE
Negative feelings	521 (23.7)	20 (25.6)
Recreation and leisure	314 (14.3)	14 (17.9)
Health care availability	205 (9.3)	8 (10.3)
Positive feelings	183 (8.3)	8 (10.3)
Mobility	176 (8)	7 (8.9)
Energy and motivation	155 (7)	4 (5.1)
Cognitive capabilities	154 (7)	5 (6.4)
Sleep and rest	114 (5.2)	3 (3.8)
Work capacity	113 (5.1)	3 (3.8)
Activities of daily living	106 (4.8)	—^e^
Financial resources	104 (4.7)	2 (2.6)
Transport	49 (2.2)	3 (3.8)
Intimacy and sex	5 (0.2)	—

^a^HRQoL: health-related quality of life.

^b^SLE: systemic lupus erythematosus.

^c^Indicates that patients were counted more than once if they discussed more than one HRQoL aspect in a post.

^d^CLE: cutaneous lupus erythematosus.

^e^Not available.

Patients frequently reported impairments in areas such as “mobility,” “cognitive capabilities,” and “recreation and leisure,” for which pain and fatigue were the leading causes. While pain primarily affected “mobility,” “recreation and leisure,” and “sleep and rest,” fatigue affected multiple HRQoL facets. Overall, the impact of pain on “mobility” and fatigue on “cognitive capabilities” was the most substantial factor impairing HRQoL (Multimedia Appendix 6).

#### Psychological Well-Being

##### Negative Feelings

Posts from 1371 patients revealed “negative feelings” (339/521, 65.1%) as the highest-ranked HRQoL facet in terms of high importance to the patient. Further examination of these posts from 521 patients revealed that severe symptoms or side effects such as pain, fatigue, rashes, and depression affected the HRQoL of these patients, thereby leading to frequent mentions of “negative feelings.”

##### Positive Feelings

“Positive feelings” were expressed by the patients who experienced feelings of satisfaction or wellness and emotions, such as excitement, interest, pride, love, and optimism. “Positive feelings” were mentioned less frequently than “negative feelings” (183/2199, 8.3% vs 521/2199, 23.7%); nevertheless, they were still considered as highly important HRQoL by 63.2% (116/183) of patients, which is comparable to the high importance of “negative feelings” (339/521, 65.1%), suggesting that patients viewed both dimensions as equally important. Among the positive emotions mentioned, “feeling better,” “thankfulness,” and “hopefulness” were the most commonly reported. Notable patient quotations on “negative feelings” and “positive feelings” are presented in Multimedia Appendix 7.

#### Physical Well-Being

##### Mobility

Patients reported that the severity of their symptoms limited physical activity and mobility. “Mobility” was mentioned in 176 patient posts corresponding to 8% of all HRQoL patient mentions (n=2199). Within this facet, approximately 58% (103/176) of patients considered this HRQoL aspect as of high importance. Primary factors impeding “mobility” (Multimedia Appendix 6) were attributed to the pain and fatigue they experienced. Moreover, patients noted that reduced mobility negatively impacted their overall health, social life, and mental well-being. One patient described “immobility” as analogous to “poor-performing robot” (Multimedia Appendix 7).

##### Activities of Daily Living

This HRQoL facet explored a person's ability to perform usual daily living activities. This facet was identified as highly important by 50.4% (53/106) of patients. Most patients described problems performing (routine) household activities, personal hygiene, going (outside) for a walk, or even getting out of bed due to pain or lack of energy. Patients’ quotations on their struggles with “activities of daily living” are provided in Multimedia Appendix 7.

##### Recreation and Leisure

For most patients (129/314, 41.3%), the HRQoL facet “recreation and leisure” was described as being highly important. Owing to the severity of the symptoms, this aspect was frequently described as being hindered, limited, or achievable only with appropriate treatments. Patients reported that joint pain, bleeding, or exhaustion frequently limited their physical activity. Additionally, patients with photosensitivity found it challenging to perform leisure activities such as outdoor sports, watching television, or just relaxation. Patients’ quotations on “recreation and leisure” are provided in Multimedia Appendix 7.

##### Role Participation

Symptoms, especially when combined with flare-ups, limited the everyday functions of patients with SLE and impacted their role participation in multiple ways. Patients reported a decrease in self-esteem and restricted social relationships. Several patients felt that they could not fulfill their parenting duties and were a burden to their partners. Moreover, patients reported that symptoms of SLE compromised their ability and capacity to work.

### Treatment Experience of Patients With SLE

#### Overview

To evaluate the treatment experiences and unmet needs of patients with SLE, the most frequently used current treatment options (nonpharmacological and pharmacological interventions) mentioned by patients were quantitatively analyzed. The level of satisfaction among patients with SLE with existing treatment options and coping mechanisms is described below.

#### Current Treatment Options

Exercise, sun protection measures, meditation, and massage were the most commonly reported nonpharmacological interventions (Table 4). Patients found that nonpharmacological interventions, particularly exercise activities such as walking, e-biking, swimming, yoga, or Pilates, and other activities such as meditation and massages to be effective coping mechanisms for dealing with the disease and its symptoms. Additionally, based on 1566 posts, hydroxychloroquine, prednisone, and methotrexate were the most frequently reported pharmacological interventions (Table 5). Negative feedback for pharmacological interventions was mostly related to the drug’s side effects (Multimedia Appendix 8).

**Table 4:** Nondrug treatments most frequently reported by patients with SLE^a^ (n=482^b^ patient mentions and n=267 patients) and CLE^c^ (n=24^b^ patient mentions from n=16 patients).

Nondrug treatments	Mentions in posts, n (%)	
	SLE	CLE
Exercise	222 (46.1)	7 (29.2)
Sun protection	82 (17)	12 (50)
Meditation	43 (8.9)	—^d^
Massage	36 (7.5)	1 (4.2)
Herbal medicine	32 (6.6)	1 (4.2)
Nutrition therapy	30 (6.2)	1 (4.2)
Acupuncture	18 (3.7)	2 (8.3)
Cognitive behavioral therapy	11 (2.3)	—
Vitamin D supplements	8 (1.7)	—

^a^SLE: systemic lupus erythematosus.

^b^Indicates that patients were counted more than once if they discussed multiple nondrug treatments in a post.

^c^CLE: cutaneous lupus erythematosus.

^d^Not available.

**Table 5:** Drug treatments most frequently reported by patients with SLE^a^ (n=1566^b^ posts) and CLE^c^ (n=82^b^ posts).

Drug treatments	Mentions in posts, n (%)	
	SLE	CLE
Hydroxychloroquine	538 (34.4)	42 (51.2)
Prednisone	322 (20.6)	8 (9.6)
Methotrexate	303 (19.3)	29 (35.4)
Azathioprine	107 (6.8)	6 (7.3)
Prednisolone	100 (6.4)	17 (20.7)
Aspirin	49 (3.1)	1 (1.2)
Ibuprofen	44 (2.8)	—^d^
Warfarin	42 (2.7)	—
Paracetamol	35 (2.2)	—
Quinacrine	26 (1.7)	7 (8.5)
Cyclosporine	—	4 (4.8)
Tacrolimus	—	3 (3.7)
Naproxen	—	2 (2.4)

^a^SLE: systemic lupus erythematosus.

^b^Indicates that patients were counted more than once they discussed more than one drug treatment in a post.

^c^CLE: cutaneous lupus erythematosus.

^d^Not available.

#### Treatment Options and HRQoL Aspects

Many relevant aspects of HRQoL, such as “recreation and leisure,” “energy and motivation,” “activities of daily living,” and “mobility,” were improved by nonpharmacological interventions, particularly exercise or meditation. Despite the undesirable side effects, patients frequently used medications, such as hydroxychloroquine, azathioprine, prednisolone, or mycophenolate mofetil, which improved SLE symptoms, although to a lesser extent than that achieved using nonpharmacological interventions. Regarding treatment effects on different aspects of HRQoL (n=5336 posts), 473 (8.9%) reported worsening, while 286 (5.4%) reported improvement of an HRQoL aspect. The most burdensome symptoms, such as pain, fatigue, and rashes, were poorly managed by available drug treatment options for patients with SLE.

#### Level of Satisfaction With Current Treatment Options

Both positive (65/286 posts) and negative (56/473 posts) feedback were documented when analyzing the level of satisfaction. The reasons for a low level of satisfaction included the lack of treatment options, failure of previously successful treatments due to side effects, or no treatment at all. A high level of satisfaction was primarily attributed to the management of symptoms, initiation of treatments following a successful diagnosis, knowledge level of the doctors treating the patients, and availability of options in case of treatment failure. The most effective pharmacological interventions included prednisone and hydroxychloroquine. However, side effects, such as allergic reactions, insomnia, and increased appetite, caused some patients to discontinue treatment. Discontinuation of treatment and patient satisfaction with drug treatments were frequently attributed to insurance coverage (such as expensive medications). Patient quotations on the levels of satisfaction with current treatment options are presented in Multimedia Appendix 8.

#### Diagnosis and Access to Care

Health care availability was frequently mentioned in 285 posts authored by 205 patients with SLE. Health care availability was considered as the most important aspect of HRQoL by 45.8% (n=94) of patients with SLE. Some patients with SLE (n=10) reported having received a misdiagnosis or a correct diagnosis only after several years of experiencing symptoms. Several patients with SLE experienced delays in diagnosis and did not receive proper treatment until they found a doctor with enough expertise to diagnose or investigate their symptoms or until they were referred to a specialist. Patients with SLE often managed and advocated for themselves by switching doctors and actively seeking specialists.

#### Coping Mechanisms

Despite their symptom-related restrictions, patients with SLE reported attempting to stay active and perform daily tasks, even if they could manage only a few of them. Reducing the number of their daily activities helped patients prevent negative side effects and flare-ups. Patients shared coping strategies such as taking medications for side effects and establishing healthy routines involving adequate sleep, proper nutrition, exercise, and hydration. A total of 28 patients (n=40 posts) reported that physical activities or exercise positively affected their physical and psychological well-being. Exercise, meditation, Tai Chi, and yoga were all reported to reduce the burden of symptoms. Some patients found that following an anti-inflammatory diet helped reduce skin and joint pain, thereby allowing them to engage in more physical activities. Patient quotations on coping mechanisms are presented in Multimedia Appendix 8.

### Disease Burden in Patients With CLE

#### Overview

The investigation of disease burden in patients with CLE was limited owing to the small sample size (106 patients with CLE and 243 patient posts). Disease burden could be quantified only for the most prevalent symptoms, areas of involvement (body parts), and sleep disturbances.

#### Patient-Reported Symptoms

Based on an analysis of 244 posts from 118 patients, the most frequently (>5% [6 patients]) reported symptoms were rash (n=27, 23%), pain (n=25, 21%), and fatigue (n=19, 16%) alopecia (n=11, 9%), arthralgia (n=10, 8%), and anxiety and scar (each n=7, 6%; Table 2). Sleep disorders were reported by a total of 8 patients in 10 posts, with insomnia (n=2, 25%) being the most common side effect of lupus medications. Notable quotations from patients describing their symptoms are presented in Multimedia Appendix 4.

#### Areas of Involvement

Based on an analysis of 169 posts from 148 patients, the skin (n=38, 26%), face (n=20, 14%), legs (n=15, 10%), and hairs (n=15, 10%) were the most frequently reported areas of involvement affected by symptoms (Multimedia Appendix 5).

### Overall HRQoL of Patients With CLE

HRQoL facets were discussed by 47 patients with CLE in 78 of 243 (32.1%) posts. The distribution of HRQoL facets of this population (n=78 posts) was similar to that of patients with SLE, with “negative feelings” (n=20, 26%) and “recreation and leisure” (n=14, 18%) being the most frequently reported dimensions of HRQoL based on the total number of posts (Table 3). Few patients mentioned that their condition negatively affected their ability to work. Although “positive feelings,” “health care access,” and “mobility” were frequently mentioned, differences in ranking could not be identified because of the small sample size (n<10) of individual HRQoL facets. Quotations of patients with CLE regarding their HRQoL are available in Multimedia Appendix 7.

### Treatment Experience of Patients With CLE

#### Overview

The treatment experiences and unmet needs of patients with CLE were quantitatively analyzed by assessing the most frequently used treatment options (pharmacological and nonpharmacological interventions) as mentioned by the patients and exploring patient experiences related to diagnostic delay, health care availability, and coping mechanisms.

#### Nonpharmacological Interventions

From 24 posts, sun protection (n=12, 50%) and exercise (n=7, 29%; Table 4) were the most frequently reported nondrug treatments. Patients found dietary changes, massages, and exercise to be helpful in managing their symptoms and flare-ups.

#### Drug Agents

The most frequently reported medications (n=119 posts) were hydroxychloroquine (n=42, 35%), methotrexate (n=29, 24%), and prednisolone (n=17, 14%; Table 5). Negative statements regarding treatment experiences were mostly related to side effects of the medications (Multimedia Appendix 8). Methotrexate was associated with side effects, such as fatigue, brain fog, and lack of sleep, which led to patients having to take time off work. Positive statements regarding pharmacological interventions were infrequent (Multimedia Appendix 8).

#### Diagnosis and Access to Care

Patients with CLE reported difficulties in receiving the correct diagnosis because health care providers did not give sufficient consideration to their skin lesion complaints. They reported being confused or misled by the recommendations of dermatologists and rheumatologists, and they received little support with an understanding of their diagnosis and the symptoms to expect from their health care professionals (HCPs). Patients reported feeling frustrated because dermatologists referred them to rheumatologists who prescribed different medications which only made their symptoms worse (Multimedia Appendix 8). Despite showing clear symptoms and positive diagnostic markers, HCPs often advised patients that they “do not have lupus” or had gone “dormant,” highlighting a concerning trend of misdiagnosis and lack of belief by HCPs when it comes to lupus and related conditions.

## Discussion

### Principal Findings

This retrospective study investigated the experiences of patients living with SLE or CLE, who publicly discussed their experiences in web-based social media platforms. Patients who were active on these platforms frequently shared and discussed their HRQoL, their perceptions of the disease burden, and treatment experiences affecting their daily lives. Pain and fatigue were identified as symptoms that most impaired HRQoL, negatively impacting physical and psychological well-being. The most frequently used pharmacological interventions included hydroxychloroquine, prednisone, and methotrexate, while nonpharmacological interventions included exercise, meditation, and proper nutrition (diet). Nonpharmacological interventions were more frequently associated with improved HRQoL.

Several studies have explored the disease burden and treatment experiences among patients diagnosed with SLE and CLE [25-31]. Qualitative studies focusing on outcomes reported by patients with SLE suggest that HRQoL is significantly impaired in these patients [26-29]. A qualitative literature analysis of 58 studies relating to the burden of SLE found that in all of these studies, the factors that appeared most frequently affecting the HRQoL in patients with SLE (based on the number of citations) were advanced age, fatigue, the coexistence of neurological or psychiatric conditions (especially depression or anxiety), limited educational achievement, and financial challenges such as poverty or low household income [26]. This is mostly consistent with the findings of this study, wherein pain and fatigue were identified as key factors negatively impacting HRQoL in patients with SLE. The severity of these symptoms affected patients’ mobility, cognitive abilities, recreation and leisure activities, and sleep and rest. Patients with SLE often found it challenging to engage in work or recreational activities such as sports, biking, or running. Moreover, these impairments had a negative impact on psychological aspects such as low self-esteem, social relationships (such as feeling like a burden to others), and role functions (such as parental responsibilities and the capacity to work).

Lupus symptoms often result in sleep disturbances, which may in turn aggravate fatigue and exhaustion. Sleep disorders in patients with SLE are often linked to disease activity, pain, and fatigue and are influenced by psychosocial and psychological factors [30]. This is consistent with the findings of this study, wherein patients with SLE or CLE reported symptoms such as pain, fatigue, and anxiety, which primarily impacted the “sleep and rest” HRQoL facet. In this study, the correlation between sleep and lupus symptoms was found to be bidirectional, that is, disrupted sleep was associated with increased fatigue and may also worsen symptoms such as pain and discomfort leading to poor sleep quality, thereby, negatively impacting the overall HRQoL. Additionally, some patients regularly experienced insomnia-related difficulties, which even lasted for years in some cases.

In this study, patients with SLE discussed worsening of HRQoL twice as much in the context of pharmacological interventions, suggesting that the most burdensome symptoms, such as pain, fatigue, and rash, were still inadequately controlled under the current treatment options. Notably, patients receiving pharmacological interventions reported positive experiences with HCPs, who considered their symptoms seriously and had adequate knowledge of lupus for providing effective treatments. They appreciated receiving treatments that helped manage their symptoms or the availability of other options if treatments failed, particularly following a successful diagnosis. In contrast, a low satisfaction level with current treatment was attributed to the lack of treatment options available, previously successful treatments that later failed, or not receiving any treatment at all. This finding highlights that effective patient-physician communication and improving patients’ knowledge about disease and treatment through patient education strategies are important for improving patients’ adherence to therapy in SLE [31].

Medications that are effective in treating lupus are frequently associated with undesirable side effects, thereby leading to patient dissatisfaction [31]. This can also be emotionally challenging for patients as it can negatively impact their HRQoL. To overcome such difficulties, patients may seek alternative nonpharmacological treatment options and associated coping strategies [32-34]. Notwithstanding the symptom-related limitations, the patients with SLE and CLE included in this study reported that they attempted to remain active and complete daily tasks despite being able to manage only a few tasks.

As observed in patients with SLE, patients with CLE experienced the same impacts on their HRQoL: “negative feelings,” “recreation and leisure,” and “health care availability.” The HRQoL of these patients with CLE was also impacted by the visibility of lesions regardless of the levels of disease activity. Skin lesions or scars that were constantly visible, alopecia, photosensitivity, and the chronic nature of the disease significantly impaired HRQoL. Patients with CLE felt that they were neglected and did not receive the necessary attention in health care settings. They highlighted that they experienced more difficulties than patients with SLE owing to misdiagnosis, delayed diagnosis, and contradictory treatment approaches between dermatologists and rheumatologists.

This study demonstrated that social listening could be useful for the generation and analysis of large amounts of data from web-based platforms, such as social media, and patient forums. The approach offers an opportunity to obtain insights from a large patient population by capturing their real-time conversations and contextual understanding of their perspectives [17]. Thus, social listening complemented with traditional methods, such as interviews, surveys, focus groups, and advisory boards or panels, can facilitate a deeper understanding of patients’ perspectives and their unmet needs. Additionally, social listening is fast, eliminates the need for patient recruitment, reduces recall bias through instant platforms, does not burden patients, and offers anonymity in reporting socially embarrassing symptoms, thereby reducing reporting bias [17,18]. We departed from traditional methods and used natural language processing and artificial intelligence in this study to apply and enhance both qualitative and quantitative methods. This facilitated more rigorous analyses of large amounts of unsolicited patient-reported narratives from multiple web-based patient forums to better understand patients' perspectives and disease burden and identify unmet medical needs.

In the context of drug development, identifying and integrating unmet patient needs and experiences in the decision-making process and evidence-generation strategies are important to ensure that patients’ perspectives and needs are considered and truly represented across the entire continuum of the process [16]. Social media offers access to difficult-to-reach populations or help to concentrate on groups with specific conditions or disorders [35,36]. By actively listening to patients’ descriptions of their challenges on social media platforms, pharmaceutical organizations could gain insight into their daily struggles and identify the most relevant and impactful factors [17]. The increasing presence of patients on social media represents an opportunity for pharmaceutical organizations to identify relevant knowledge for different stages of drug development, with a focus on patients’ HRQoL and beyond.

### Limitations

This study had several limitations. First, this study relied on publicly available social media data, raising concerns regarding the accuracy of self-diagnosis or self-reported patient experience. Patients who opted not to publicly share their profiles (and were therefore excluded from the study) may have different opinions, thereby resulting in potential bias. Second, the data set may contain duplicate author profiles, wherein similar authors could have been active on multiple social media platforms. Third, this study only included English-speaking countries and younger populations, which could be a source of bias. Fourth, this study did not examine differences across subgroups (patient populations with SLE and CLE) owing to the inability to identify race or ethnicity or other sociodemographic variables. Fifth, the analysis of patients with CLE was limited by a small sample size, which is why several outcomes reported for SLE could not be reported for CLE; therefore, the results regarding CLE should be interpreted cautiously. The authors’ opinion is that similar type of analyses should be conducted in the future for other novel pharmacological agents to gather valuable insights into the experiences of patients while living with the disease.

### Conclusions

This social listening study sheds light on the experiences of patients living with SLE and CLE active on social media, providing valuable insight into their experiences, which have not been extensively investigated. SLE and CLE affect all aspects of patients’ lives owing to their wide-ranging manifestations. This study showed varying severity and frequency of symptoms that were reported as burdensome by patients. Moreover, several aspects of HRQoL and factors contributing to HRQoL improvement or worsening were discussed by patients. The results of this study suggest that current treatment options provide insufficient relief, thereby warranting the development of more effective treatments with good tolerability and safety to address the heavy disease burden and unmet needs of patients with SLE and CLE. Our findings can serve as a valuable resource to inform and shape activities and decisions in drug development to meet patients’ needs and improve their HRQoL.

## Appendix

Multimedia Appendix 1 List of relevant web-based social media sources retrieved from third-party social media data provider “SocialGist”.

Multimedia Appendix 2 Glossary of terms.

Multimedia Appendix 3 Personal data protection measures.

Multimedia Appendix 4 Quotations of patients with systemic lupus erythematosus or cutaneous lupus erythematosus describing their symptoms.

Multimedia Appendix 5 Most frequently mentioned body parts by patients with systemic lupus erythematosus or cutaneous lupus erythematosus.

Multimedia Appendix 6 Heatmap showing the impairment (negative impact) of a symptom on an aspect of health-related quality of life in patients with systemic lupus erythematosus (n=159 patients from 716 posts).

Multimedia Appendix 7 Quotations of patients with systemic lupus erythematosus or cutaneous lupus erythematosus on their health-related quality of life.

Multimedia Appendix 8 Quotations of patients with systemic lupus erythematosus and cutaneous lupus erythematosus on their drug treatment experiences.

## Abbreviations

CLE: cutaneous lupus erythematosus
HCP: health care professional
HRQoL: health-related quality of life
SLE: systemic lupus erythematosus

## References

[1] Vaillant AAJ, Goyal A, Varacallo M (2022). Systemic Lupus Erythematosus.

[2] García-Carrasco M, Mendoza PC, Solís PJC, Morales IE, Cervera R, Anaya JM, Rojas-Villarraga A, Anaya JM, Cervera R, Levy RA, Shoenfeld Y (2013). Systemic lupus erythematosus. Autoimmunity: From Bench To Bedside.

[3] Systemic lupus erythematosus. Centers for Disease Control and Prevention.

[4] Wenzel J (2019). Cutaneous lupus erythematosus: new insights into pathogenesis and therapeutic strategies. Nat Rev Rheumatol.

[5] Cooper EE, Pisano CE, Shapiro SC (2021). Cutaneous manifestations of "lupus": systemic lupus erythematosus and beyond. Int J Rheumatol.

[6] Lupus in women. Centers for Disease Control and Prevention.

[7] Cutaneous lupus (skin lupus). Cleveland Clinic.

[8] Robinson D, Aguilar D, Schoenwetter M, Dubois R, Russak S, Ramsey-Goldman R, Navarra S, Hsu B, Revicki D, Cella D, Rapaport MH, Renahan K, Ress R, Wallace D, Weisman M (2010). Impact of systemic lupus erythematosus on health, family, and work: the patient perspective. Arthritis Care Res (Hoboken).

[9] Klein R, Moghadam-Kia S, Taylor L, Coley C, Okawa J, LoMonico J, Chren MM, Werth VP (2011). Quality of life in cutaneous lupus erythematosus. J Am Acad Dermatol.

[10] Tian J, Zhang D, Yao X, Huang Y, Lu Q (2023). Global epidemiology of systemic lupus erythematosus: a comprehensive systematic analysis and modelling study. Ann Rheum Dis.

[11] Durosaro O, Davis MDP, Reed KB, Rohlinger AL (2009). Incidence of cutaneous lupus erythematosus, 1965-2005: a population-based study. Arch Dermatol.

[12] Katarzyna PB, Wiktor S, Ewa D, Piotr L (2023). Current treatment of systemic lupus erythematosus: a clinician's perspective. Rheumatol Int.

[13] Thong B, Olsen NJ (2017). Systemic lupus erythematosus diagnosis and management. Rheumatology (Oxford).

[14] Touma Z, Gladman DD (2017). Current and future therapies for SLE: obstacles and recommendations for the development of novel treatments. Lupus Sci Med.

[15] Leung J, Kloos L, Kim AH, Baker EA (2021). Development of a digital toolkit to improve quality of life of patients with systemic lupus erythematosus. Digit Health.

[16] U.S. Department of Health and Human Services, Food and Drug Administration, Center for Drug Evaluation and Research (CDER), Center for Biologics Evaluation and Research (CBER) (2022). Patient-focused drug development: methods to identify what is important to patients: guidance for industry, food and drug administration staff, and other stakeholders. U.S. Food and Drug Administration.

[17] Schmidt AL, Rodriguez-Esteban R, Gottowik J, Leddin M (2022). Applications of quantitative social media listening to patient-centric drug development. Drug Discov Today.

[18] Humphrey L, Willgoss T, Trigg A, Meysner S, Kane M, Dickinson S, Kitchen H (2017). A comparison of three methods to generate a conceptual understanding of a disease based on the patients' perspective. J Patient Rep Outcomes.

[19] Spies E, Andreu T, Koelling J, Hartung M, Kamudoni P, Park J (2022). PCR251 retrospective social listening study of patients living with Systemic Lupus Erythematosus (SLE): understanding the patient experience. Value Health.

[20] Spies E, Andreu T, Koelling J, Hartung M, Kamudoni P, Park J (2022). PCR18 an exploratory retrospective social listening study to identify patient experiences associated with cutaneous lupus erythematosus (CLE). Value Health.

[21] Tadmouri A, Alivon M, Andreu T, Hartung M, Ryll B, Rauch G, Kiecker F, Cimiano P (2022). RWD141 exploration of melanoma patient-generated real-world data using an AI-based social listening approach. Value Health.

[22] Hartung M, Schwering N, Loonus Y, Cimiano P, Jaeger AA, Collins B (2021). Automatically analyzing online patient experience data with natural language processing: an instrument to investigate health status and help-seeking factors in patients with obesity. Qual Life Res.

[23] WHOQOL: measuring quality of life. World Health Organization.

[24] Moreno MA, Goniu N, Moreno PS, Diekema D (2013). Ethics of social media research: common concerns and practical considerations. Cyberpsychol Behav Soc Netw.

[25] Holloway L, Humphrey L, Heron L, Pilling C, Kitchen H, Højbjerre L, Strandberg-Larsen M, Hansen BB (2014). Patient-reported outcome measures for systemic lupus erythematosus clinical trials: a review of content validity, face validity and psychometric performance. Health Qual Life Outcomes.

[26] Schmeding A, Schneider M (2013). Fatigue, health-related quality of life and other patient-reported outcomes in systemic lupus erythematosus. Best Pract Res Clin Rheumatol.

[27] Pettersson S, Lövgren M, Eriksson LE, Moberg C, Svenungsson E, Gunnarsson I, Henriksson EW (2012). An exploration of patient-reported symptoms in systemic lupus erythematosus and the relationship to health-related quality of life. Scand J Rheumatol.

[28] Kernder A, Elefante E, Chehab G, Tani C, Mosca M, Schneider M (2020). The patient's perspective: are quality of life and disease burden a possible treatment target in systemic lupus erythematosus?. Rheumatology (Oxford).

[29] Sterling K, Gallop K, Swinburn P, Flood E, French A, Al Sawah S, Iikuni N, Naegeli A, Nixon A (2014). Patient-reported fatigue and its impact on patients with systemic lupus erythematosus. Lupus.

[30] Palagini L, Tani C, Mauri M, Carli L, Vagnani S, Bombardieri S, Gemignani A, Mosca M (2014). Sleep disorders and systemic lupus erythematosus. Lupus.

[31] Farinha F, Freitas F, Águeda A, Cunha I, Barcelos A (2017). Concerns of patients with systemic lupus erythematosus and adherence to therapy—a qualitative study. Patient Prefer Adherence.

[32] Fangtham M, Kasturi S, Bannuru RR, Nash JL, Wang C (2019). Non-pharmacologic therapies for systemic lupus erythematosus. Lupus.

[33] Cornet A, Mazzoni D, Edwards A, Monzani D, Pravettoni G, Andersen J, Mosca M (2022). Coping with systemic lupus erythematosus in patients' words. Lupus Sci Med.

[34] Case S, Sinnette C, Phillip C, Grosgogeat C, Costenbader KH, Leatherwood C, Feldman CH, Son MB (2021). Patient experiences and strategies for coping with SLE: a qualitative study. Lupus.

[35] Topolovec-Vranic J, Natarajan K (2016). The use of social media in recruitment for medical research studies: a scoping review. J Med Internet Res.

[36] Rastegar-Mojarad M, Liu H, Nambisan P (2016). Using social media data to identify potential candidates for drug repurposing: a feasibility study. JMIR Res Protoc.

