# Supplementary material for: Exploring the Perspectives of Patients Living With Lupus: Retrospective Social Listening Study
Source: JMIR Form Res. 2024 Feb 2;8:e52768. doi: 10.2196/52768 (PMC10873798; doi:10.2196/52768)
Supplement: Multimedia Appendix 1 [file formative_v8i1e52768_app1.doc]

###

| **Social media sources** |
| --- |
|  |
| www.healthunlocked.com |
| www.inspire.com |
| www.lupusresearch.org |
| www.healingwell.com |
| www.mumsnet.com |
| www.carenity.co.uk |
| www.medhelp.org |
| www.patient.info |
| www.dailystrength.org |
| www.whattoexpect.com |
| www.healthboards.com |
| www.community.babycenter |
| www.smartpatients.com |
