# Supplementary material for: Exploring the Perspectives of Patients Living With Lupus: Retrospective Social Listening Study
Source: JMIR Form Res. 2024 Feb 2;8:e52768. doi: 10.2196/52768 (PMC10873798; doi:10.2196/52768)
Supplement: Multimedia Appendix 2 [file formative_v8i1e52768_app2.doc]

Algorithmic coding: The process of automatically annotating patient records with patient experience concepts.

Annotation: The process of labeling sequences of text with semantic tags that assign machine-readable or human-interpretable meaning to these sequences.

Classification: consists of assigning a categorical output tag (“authored by a patient with lupus” in the context of this research) to the input data of a particular type. In the context of this research, classification is done through machine learning approaches.

Knowledge graph: A database that uses a graph structure to store knowledge about concepts and relations between them. Each concept in the graph is minimally defined as a uniquely identifiable object and member of a particular class (e.g., the concept “lupus” being a member of the class “Disease”). Concepts can be further specified in terms of typed relations to other concepts (e.g., “lupus” can be linked to the concept “butterfly rash” via a relation “has-symptom”). For each concept, different expressions that can be used to refer to this concept in natural language (e.g., synonyms or abbreviations) are stored as well.

Machine learning: A group of computational approaches to algorithmic classification based on sets of input data, generally large enough to be split into training and test data. Classification is made by a dedicated algorithm after being trained to learn the relevant classification criteria from samples of training data, that -in the context of this research- are manually annotated with the correct or expected decision by human experts. The specific nature of the relation between input and output data determines the problem class. In the context of this study, machine learning approaches were used for training NLP models for classification problems.

Natural language processing (NLP): A computational approach for making the meaning of natural language text or speech interpretable for machines. This is achieved either by mapping entire sequences of text into machine-readable representations or by extracting the most important pieces of information from a text and storing them as machine- or human-readable data.

Patient experience concepts: A means to group equivalent natural language expressions referring to aspects of the patient experience into a coherent class (e.g., “my limbs hurt” and “my leg aches” both refer to the concept of “pain”).

Patient record: The collection of all documents authored by a unique patient in an included social media site. Through algorithmic coding, the raw text contained in each patient record is automatically annotated with patient experience concepts.

Semalytix Pharos®: A patient experience research platform developed by Semalytix. In this study, several upstream processing steps were used to compute relevant results about patient-reported experiences regarding living with lupus via algorithmic coding of patient records obtained from social media. The Pharos® platform was used to visualize these results and make them accessible for further quantitative and qualitative analyses by human experts.

SocialGist: A third-party data provider used in this study to gain programmatic access to a universe of online social media sources through an application programming interface. Subsequently, tailored algorithms were used to filter and aggregate the raw data thus acquired for this study.
