# Supplementary material for: Exploring the Perspectives of Patients Living With Lupus: Retrospective Social Listening Study
Source: JMIR Form Res. 2024 Feb 2;8:e52768. doi: 10.2196/52768 (PMC10873798; doi:10.2196/52768)
Supplement: Multimedia Appendix 3 [file formative_v8i1e52768_app3.doc]

Semalytix GmbH processed personal data in a GDPR-compliant manner and with the special focus on legality of data processing and to the best efforts possible regarding the transparency for data subjects. All data processing followed a privacy by design and default approach and were performed in a fully anonymized way for processing.

These processes are based on the following key principles, among others:

- All patient data are anonymized before being further processed. Access to non‑anonymized raw data is granted only for a minimum number of technical staff (following the principle of least privilege) and kept strictly separate from all parties involved in data analysis or annotation.
- Only data sources which are publicly accessible (without password protection or other means of access restriction in place) are processed in the first place; hence, it can be assumed that all information being analyzed has been manifestly made public by the authors.
- No data integration across sources is performed. Results are evaluated and made accessible in an aggregated manner only.
- Since providing information about data processing to the data subjects directly would involve a disproportionate effort in the context of social listening, a Data Processing Impact Assessment (DPIA) was done and is reviewed regularly to demonstrate that Semalytix strictly follows the fundamental GDPR requirements with respect to lawfulness, fairness, transparency, data minimization, purpose limitation, storage limitation, accuracy, integrity, confidentiality, and accountability.
- Semalytix makes sure that data subjects can follow their rights by contacting Semalytix directly (via [privacy@semalytix.com](mailto:privacy@semalytix.com)). In addition, contractual obligations with data providers are in place to forward any data subject inquiries they receive.
- Semalytix has established technical and organizational measures to address and minimize the risks for the data subject.
- Based on a privacy by design and default approach, Semalytix ensures that conclusions on and relations to individual data subjects are not possible within any service offered by Semalytix, and the data processing does not result in any impact on data subjects.
