# Supplementary material for: Exploring the Perspectives of Patients Living With Lupus: Retrospective Social Listening Study
Source: JMIR Form Res. 2024 Feb 2;8:e52768. doi: 10.2196/52768 (PMC10873798; doi:10.2196/52768)
Supplement: Multimedia Appendix 4 [file formative_v8i1e52768_app4.doc]

| **Disease Burden on Patients (Patient-reported Symptoms)** | |
| --- | --- |
| **SLE** | |
|  | |
| Pain, fatigue and rash | Pain: “I have pain in every bit of my body.”  Fatigue: “I feel a lot of fatigue even if I sleep 10 hours a day.”  Rash: “I found myself in bed, crying from the discoid rash that was all over my body.” |
| Anxiety | “For the last two years I have felt out of control, scared and I've been getting increasingly sick.” |
| Depression | “I still experience the brain fog, ear and skin issues, chest pain, depression, joint and muscle pain, and some days it's hard to even get out of bed.” |
| Photosensitivity | “I am stuck at home behind closed curtains. It's very frustrating. It's the hardest part of lupus for me because it's so isolating.”  “My house is dark.”  “I am EXTREMELY sun and UV sensitive. (fluorescent lights, too) Any trips, even at 9am for a short run to a doctor, causes a flare-up with itching everywhere.”  “Lightest touch felt like my skin was on fire.”  “I use sunblock as a part of my beauty routine.” |
| Alopecia | “My hair was falling off in chunks. My bald spot has hair now thank God! I have to work and do school at the same time. He said it was alopecia. (…) “ |
| Sleep disorder | “I have had sleeping issues for over 2 years now and some days I cannot sleep for 48 hours straight.”  “Mostly this week I've had insomnia every night.”  “I've noticed during the last 1.5 to 2 years when I have insomnia a flare, usually in 24–48 hours, is soon to follow.” |
| Medication related insomnia | “I have been taking hydroxychloroquine for my lupus for months now, and it has been great, but the insomnia is crazy.”  “I've been on prednisone for up to 6 months to get my symptoms under control. It always causes facial acne, increased appetite, and insomnia.” |
| Ways to mitigate sleep maintenance problems | “Gentle exercise helps a lot and walking is really good. I also use extra strength Tylenol® and a muscle relaxer called Baclofen® at night to help me sleep.”  “I am continuing with acupuncture because it has consistently been helping with all of my lupus symptoms (rhinitis, sinusitis, insomnia, and back pain).” |
| **CLE** | |
|  | |
| Rashes | “I was diagnosed with subacute cutaneous lupus (SCLE) in 2017 and at the time my hands and feet were in a terrible state with chronic ulceration. The lesions with me however are usually on the palm of my hands and soles of my feet. I get a burning feeling beforehand and my skin becomes very red and inflamed.. yes hot hot hot!! I can't really say that they blister as such but the skin swells and then splits and I do get them on all of my fingers.in the cracks on the underside and down the sides of my fingers!!” |
| Photosensitivity | “I treat the sun as an allergy, certainly until it was under control. So, in addition to sunblock, I cover up…hat, gloves, sun jacket if summertime, sun up shirts, etc. Don't sit outside in middle of day, watch out for reflective light etc.” |
| Sleep disorder | “The pain is so bad I can barely sleep.”  “…and I cannot sleep at all and feel so fatigued during the day. I feel too tired to join my run group this evening. I am taking a sleeping tablet plus paracetamol and ibuprofen most nights.” |

CLE, cutaneous lupus erythematosus; SLE, systemic lupus erythematosus
