# Supplementary material for: Exploring the Perspectives of Patients Living With Lupus: Retrospective Social Listening Study
Source: JMIR Form Res. 2024 Feb 2;8:e52768. doi: 10.2196/52768 (PMC10873798; doi:10.2196/52768)
Supplement: Multimedia Appendix 5 [file formative_v8i1e52768_app5.docx]

Body parts most frequently reported by patients with SLE (n=1778^a^ patients from 2443 posts) and CLE (n=148^a^ patient mentions from 169 posts).

| **Body parts** | **Proportion of patients, % (n)** | |
| --- | --- | --- |
|  | **SLE** | **CLE** |
| Skin | 12.6 (224) | 26 (38) |
| Face | 12.6 (224) | 14 (20) |
| Eye | 11.1 (197) | 9 (13) |
| Foot | 10.3 (184) | 8 (12) |
| Leg | 10.3 (184) | 10 (15) |
| Head | 10 (178) | 5 (8) |
| Heart | 8.9 (159) | - |
| Mouth | 8.3 (148) | - |
| Kidney | 8.1 (144) | - |
| Hair | 11.1 (197) | 10 (15) |
| Thorax | - | 7 (10) |
| Neck | - | 6 (9) |
| Hand | - | 5 (8) |
| ^a^Indicates that patients were counted more than once if they discussed more than one body part in a post.  CLE: cutaneous lupus erythematosus; SLE: systemic lupus erythematosus. | | |
