# Supplementary figures and images for: Exploring the Perspectives of Patients Living With Lupus: Retrospective Social Listening Study
Source: JMIR Form Res. 2024 Feb 2;8:e52768. doi: 10.2196/52768 (PMC10873798; doi:10.2196/52768)

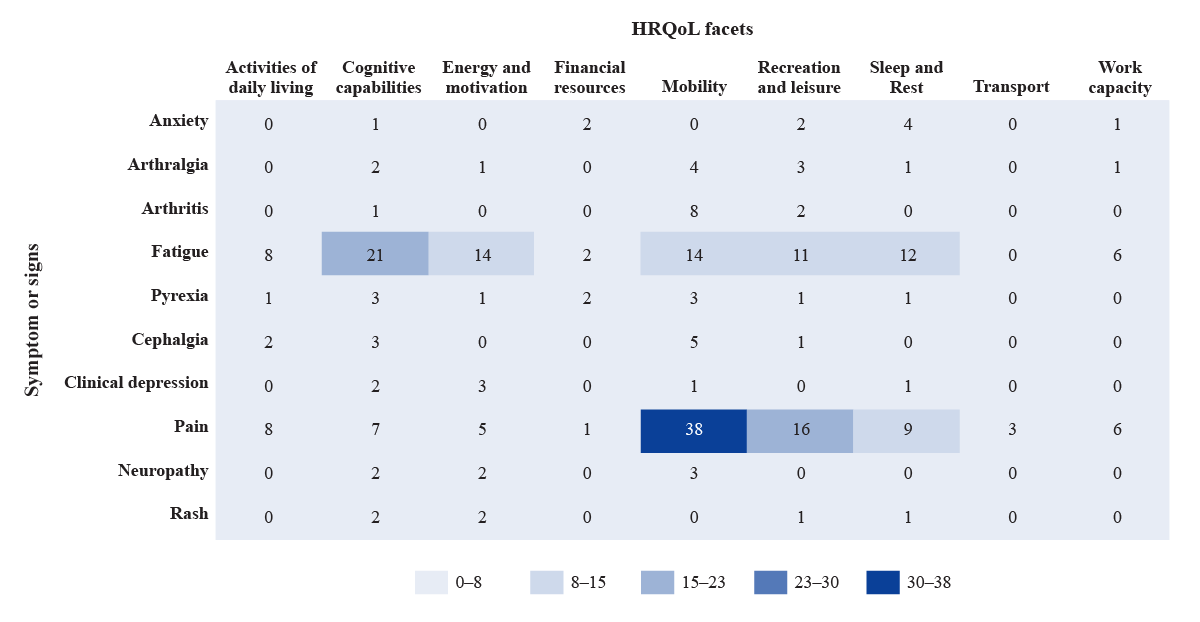

Supplement: Multimedia Appendix 6 [file formative_v8i1e52768_app6.png]
