# Supplementary material for: Exploring the Perspectives of Patients Living With Lupus: Retrospective Social Listening Study
Source: JMIR Form Res. 2024 Feb 2;8:e52768. doi: 10.2196/52768 (PMC10873798; doi:10.2196/52768)
Supplement: Multimedia Appendix 7 [file formative_v8i1e52768_app7.doc]

| **SLE** | |
| --- | --- |
|  | |
| Negative feelings | “An illness like Lupus is not a game or a simple cold or flu. Lupus kills and reduces our quality of life so much that we cannot shower, walk, have breakfast, or even sleep sometimes.”  “My entire body hurts. Every morning I wake up and find it harder and harder to get out of bed. The depression that comes along with lupus and my hideous rash is horrible. My self-esteem is nonexistent. I look absolutely hideous.”  “I feel like I randomly get angry about this whole process about everything to do with this. I’m angry my doctors won’t diagnose me (i’ve been struggling for a year and a half now with positive ANAs, all  lupus symptoms including butterfly rash). I’ve had every single lupus symptom in the book and i’m getting worse as time goes on with frequent flares.” |
| Positive feelings | “But I feel physically better now than I have at any time in the last 18 years and as long as I follow the rule to take it /medication) away from food I have no side effects.”  “Thankfully, I am feeling some better today. Pain level has come down …”  “This forum has helped me so much; You re-assured me, in addition to _USERNAME_! I’m glad I found and joined this group!”  “Joining Lupus UK 10 years ago was one of THE BEST things I’ve done in my 66 years living with lupus. You are a WONDERFUL support group organisation...and I’m thankful for you EVERY day.” |
| Activities of daily living | “Before Lupus I was an outdoor hobbies person and it’s taken years to get over that.”  “Two days ago I woke with excruciating pain in my shoulders, upper arms forearms, wrists hands and fingers which has rendered me useless in daily living activities.” |
| Mobility | “My occasional inability to make it up a set of stairs, or basically becoming a poor-performing robot who moves very, very slowly and can't operate any small digits (fingers, toes, etc)” |
| Recreation and Leisure | “I went from only being able to do one to three light things a day to being able to go throughout the whole day, most days: cleaning the house, going to the park or swimming, and running an errand or two maybe in the evening.” |
| Healthcare availability | “Eventually diagnosed with lupus and, most recently with psoriatic arthritis, the last five rheumatologists missed that, although it was very clear (even with visible evidence) if any had chosen to look.” |
| **CLE** | |
|  | |
| Daily activity and Recreation and Leisure | “I get tired easily but try and keep active by exercising regularly. When I get any sort of bug it takes me ages to clear, and I get really anxious and panicky when I’m ill. I developed horrendous heat rash in the sun so cannot sit out in the sun without factor 50 and a hat and long sleeves.” |
| Work capacity impairment | “Where do I go from here? I am unwell on a daily basis and close to losing my job as the fatigue I'm experiencing is so severe I can't get out of bed some days.” |
| Skin lesion not taken seriously by HCP | “I have mostly cutaneous lupus, had many positive ANAs, and borderline Advise tests. We moved recently and I had to find a new rheumy. Even though I had two skin biopsies that three dermapathologists studied, with a diagnosis of cutaneous lupus or dermatomyositis, the new rheumy said she thought it was fibromyalgia. …I have also had a dermatologist yell at me that ‘You don't have lupus!’ I talked back to him, and asked what he thought I had, then went out to car and cried. It is just all beyond frustrating! The disease is hard enough to manage, but doctors not believing you makes it so much harder…” |

ANA, antinuclear antibody; CLE, cutaneous lupus erythematosus; HCP, healthcare professional; SLE, systemic lupus erythematosus; UK, United Kingdom.
