# Supplementary material for: Exploring the Perspectives of Patients Living With Lupus: Retrospective Social Listening Study
Source: JMIR Form Res. 2024 Feb 2;8:e52768. doi: 10.2196/52768 (PMC10873798; doi:10.2196/52768)
Supplement: Multimedia Appendix 8 [file formative_v8i1e52768_app8.doc]

| **SLE** | |
| --- | --- |
|  | |
| Negative statements | “I retried methotrexate again and it had to be stopped due to raised liver enzymes again. So, this med for me has been ruled out now. I then tried sulfasalazine in August. I managed to get up to two tablets two weeks into it. Sadly, that raised my liver enzymes to 342!! So that was then stopped.” |
|  | “This particular rheumatologist was even worse than the last. He informed me I did not have any kind of illness to be worried about. He was not convinced I was in any significant pain, and he also informed me that “even if I did have lupus” his treatment plan would not change.” |
| Positive statements | “I will look further into the good lupus doctor's work and keep my fingers crossed that the hydroxychloroquine will keep me in check for as long as possible, although tbh I hate taking the stuff.” |
|  | “I was treated with 60 mg of prednisone for months. It made me overweight, but it brought relief. …My meds also included plaquenil and immuran (added in 2004 after retention of fluid in the feet). These work perfectly for me. My lupus is well managed with Plaquenil, and I have not had any life-threatening concerns.” |
| Levels of satisfaction (low) with current treatment options | “My rheumatologist has in the past dismissed it as nothing to do with my lupus as my bloods are all fine. So, I am now left with no treatment for 6 months.”  “I retried methotrexate again and it had to be stopped due to raised liver enzymes again. So, this med for me has been ruled out now. I then tried in August sulfasalazine. I managed to get up to 2 tablets 2 weeks into it. Sadly, that raised my liver enzymes to 342!! So that was then stopped.”  “I was referred to Rheumatology at our local hospital. After discussion, it was decided to not give me any medication - just 'see how it goes.”  “This particular rheumatologist was even worse than the last. He informed me I did not have any kind of illness to be worried about. He was not convinced I was in any significant pain, and he also informed me that “even if i did have lupus” his treatment plan would not change.)” |
| Diagnosis and access to care | “I was diagnosed with lupus at age 48, but assuming I have had it for 20 years due to all of my symptoms combined over the years.”  “Eventually diagnosed with Lupus and, most recently with psoriatic arthritis (the last five rheumatologists missed that, although it was very clear, even with visible evidence) if any had chosen to look see.” |
| Coping mechanisms | Physical activities or exercise:  “Exercise - Tai Chi, Yoga helps to Relax body and mind.”  “I exercise regularly to keep my body strong and keep as active as I  can (and to prevent osteoporosis from my prednisone).”  “For me stretching helps and swimming and even just walking in a pool.”  “The other is do consider increasing exercise, it seems counter intuitive, but I have found that despite the fatigue and pain, doing some non-weight bearing exercise e.g., yoga or Pilates has helped.”  Diets (anti-inflammatory):  “I have also gone an anti-inflammatory diet and that seems to have helped my weight and my general wellbeing.”  “I have done the same thing with diet change. I have cut out foods  that cause inflammation and I eat a lot of superfoods to fight inflammation.” |
| **CLE** | |
|  | |
| Negative statements | “Hi, I am on a xathioprine 100 mg alongside a biologic drug for RA and Discoid lupus. I started it approx 2 years ago. From memory I had some nausea in the first couple of weeks and no side effects since. I have four weekly blood tests. All these drugs are a worry, but I look at it from the view of quality of life. Take precautions to avoid infection and keep on top of your blood checks. My discoid lupus is the spanner in the works and if that flares up badly, I will have to switch to mylophenate. Be careful in the sun too as it can cause sun sensitivity x.” |
|  | “Hi, I have skin lupus for 3 years now without remission.  I have tried lots of medications without success. On methotexrate for 7 months now with lots of side effects. Fatigue, nausea, and brain fog are hardest to handle. I also have Sjogrens and take mepacrine, which helps with symptoms. Anyone with lupus and Sjogrens and how to manage both medications?  I would like to return to work but with brain fog and memory loss it is not possible just now. Thank you.” |
| Daily activity and  Recreation &  Leisure | “I get tired easily but try and keep active by exercising regularly. When I get any sort of bug it takes me ages to clear, and I get really anxious and panicky when I’m ill. I develop horrendous heat rash in the sun so cannot sit out in the sun without factor 50 and a hat and long sleeves.” |
| Work capacity  impairment | “I was forced to retire from teaching which I loved. With these crazy autoimmune diseases, you live an unpredictable life, but with time hopefully you will do better.”  “Where do I go from here? I am unwell on a daily basis and close to losing my job as the fatigue I'm experiencing is so severe I can't get out of bed some days.” |
| Positive statements | “I changed my diet (stopped eating sugar and refined carbs and up my healthy fat intake. Have you heard of Healthy Keto and Intermittent fasting? I learned from Dr. _NAME_ on YouTube. This helps tackle inflammation). Also, water (I was constipated a bit probably). I am doing exercises, tummy massages and relaxation techniques. It all helps.”  “I did yoga, water aerobics, attended concerts, church, took walks in the beach area close to my house. I did anything I could think of to manage my stress. It is my belief that whether you are a laid back person will make a difference.” |
| Diagnosis and access to care | "Hi everyone, I was diagnosed with Cutaneous lupus a few years ago and was on medication for a while but have been off it now for some time as they advised my lupus had gone ‘dormant’. I have always received very little support with understanding my diagnosis and the symptoms to expect. More regularly now, I am becoming extremely tired, achy and appear to get ulcers in my nose, it can get in the way of my daily living, and I am getting quite frustrated and upset…”  “Hi there, in December last year my Dermatologist diagnosed me with urticarial vasculitis and clinical subacute cutaneous lupus, and I started on Hydroxychloroquine. This medication appeared to make my symptoms worse and so I stopped taking it, my Dermatologist then referred me to a Rheumatologist so I could be prescribed different medication that she was unable to prescribe. I waited 11 months for the appointment (which was this morning) only to be told that although I have all the symptoms to match a Lupus diagnosis my ANA levels were not positive and so there  was nothing he could do for me and told me I don't have Lupus! I queried his decision, and he was so rude, dismissed me and told me to go back to my GP to get another referral to a different Rheumatologist...” |

ANA, antinuclear antibody; CLE, cutaneous lupus erythematosus; SLE, systemic lupus erythematosus; RA, rheumatoid arthritis.
